# Clinical, biochemical, and genetic features of four patients with short‐chain enoyl‐CoA hydratase (ECHS1) deficiency

**DOI:** 10.1002/ajmg.a.38658

**Published:** 2018-03-25

**Authors:** Patricia E. Fitzsimons, Charlotte L. Alston, Penelope E. Bonnen, Joanne Hughes, Ellen Crushell, Michael T. Geraghty, Martine Tetreault, Peter O'Reilly, Eilish Twomey, Yusra Sheikh, Richard Walsh, Hans R. Waterham, Sacha Ferdinandusse, Ronald J. A. Wanders, Robert W. Taylor, James J. Pitt, Philip D. Mayne

**Affiliations:** ^1^ Department of Paediatric Laboratory Medicine Temple Street Children's University Hospital Dublin Ireland; ^2^ Wellcome Centre for Mitochondrial Research, Newcastle University, Newcastle upon Tyne, NE2 4HH United Kingdom; ^3^ Department of Molecular and Human Genetics Baylor College of Medicine Houston Texas; ^4^ National Centre for Inherited Metabolic Disorders, Temple Street Children's University Hospital Dublin Ireland; ^5^ Children's Hospital of Eastern Ontario Research Institute, University of Ottawa Ottawa Ontario Canada K1H 8L1; ^6^ Department of Human Genetics McGill University Montreal, Québec Canada H3A 1B1; ^7^ Department of Radiology Temple Street Children's University Hospital Dublin Ireland; ^8^ Laboratory Genetic Metabolic Diseases, Department of Clinical Chemistry Academic Medical Center Amsterdam The Netherlands; ^9^ Victorian Clinical Genetics Services, Murdoch Children's Research Institute Melbourne Australia

**Keywords:** 3‐methylglutaconate, ECHS1, Leigh syndrome, SCEH deficiency, Valine

## Abstract

Short‐chain enoyl‐CoA hydratase (SCEH or ECHS1) deficiency is a rare inborn error of metabolism caused by biallelic mutations in the gene *ECHS1* (OMIM 602292). Clinical presentation includes infantile‐onset severe developmental delay, regression, seizures, elevated lactate, and brain MRI abnormalities consistent with Leigh syndrome (LS). Characteristic abnormal biochemical findings are secondary to dysfunction of valine metabolism. We describe four patients from two consanguineous families (one Pakistani and one Irish Traveler), who presented in infancy with LS. Urine organic acid analysis by GC/MS showed increased levels of *erythro*‐2,3‐dihydroxy‐2‐methylbutyrate and 3‐methylglutaconate (3‐MGC). Increased urine excretion of methacrylyl‐CoA and acryloyl‐CoA related metabolites analyzed by LC‐MS/MS, were suggestive of SCEH deficiency; this was confirmed in patient fibroblasts. Both families were shown to harbor homozygous pathogenic variants in the *ECHS1* gene; a c.476A > G (p.Gln159Arg) *ECHS1*variant in the Pakistani family and a c.538A > G, p.(Thr180Ala) *ECHS1* variant in the Irish Traveler family. The c.538A > G, p.(Thr180Ala) *ECHS1* variant was postulated to represent a Canadian founder mutation, but we present SNP genotyping data to support Irish ancestry of this variant with a haplotype common to the previously reported Canadian patients and our Irish Traveler family. The presence of detectable *erythro*‐2,3‐dihydroxy‐2‐methylbutyrate is a nonspecific marker on urine organic acid analysis but this finding, together with increased excretion of 3‐MGC, elevated plasma lactate, and normal acylcarnitine profile in patients with a Leigh‐like presentation should prompt consideration of a diagnosis of SCEH deficiency and genetic analysis of *ECHS1*. ECHS1 deficiency can be added to the list of conditions with 3‐MGA.

## 1 INTRODUCTION

Biallelic mutations in the *ECHS1* gene (OMIM 602292) lead to a deficiency of short‐chain enoyl‐CoA hydratase (SCEH/crotonase EC4.2.1.17) resulting in a rare inborn error of valine metabolism (Haack et al., 2015; Peters et al., 2014; Sakai et al., 2015; Tetreault et al., 2015). Clinical presentation includes infantile‐onset of severe developmental delay and regression and seizures, with elevated plasma lactate and brain MRI abnormalities consistent with Leigh syndrome (LS) as described by Denis Leigh in 1951 (Leigh, 1951) and more recently by Baertling et al. in 2014 (Baertling, 2014). Genetic diagnosis of SCEH deficiency can be complicated by the clinical, radiological, and biochemical overlap with mitochondrial encephalopathic disorders; often occurring with respiratory chain complex deficiencies (Ferdinandusse et al., 2015; Sakai et al., 2015) or pyruvate dehydrogenase (PDH) deficiency (Bedoyan et al., 2017; Ferdinandusse et al., 2015; Peters et al., 2014).

**Figure 1:**
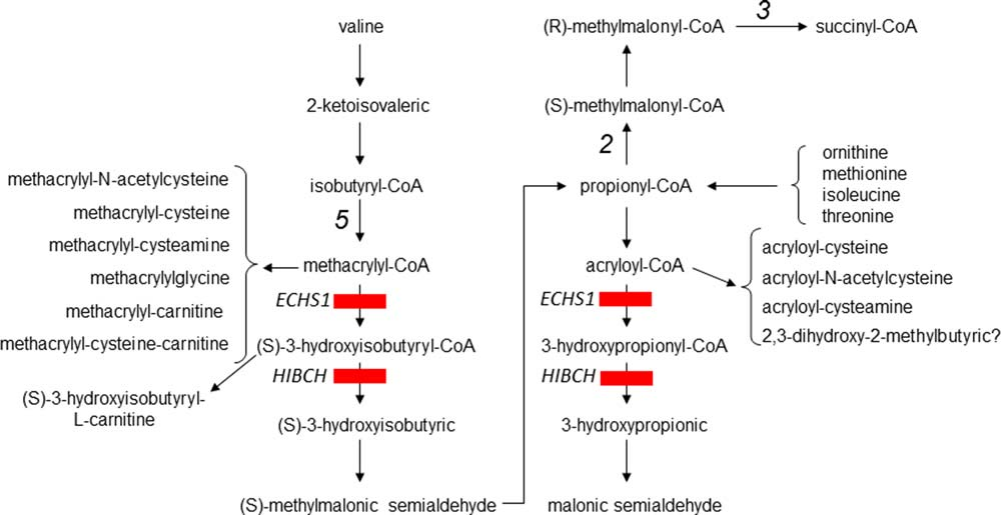
Valine catabolic pathway showing formation of metabolites listed in Table 4, which are abnormal in SCEH and HIBCH deficiency. The metabolic origin of 2,3‐dihydroxy‐2‐methylbutyrate is currently unclear. Enzymes are numbered: 2 = propionyl‐CoA carboxylase; 3 = (R)‐methylmalonyl‐CoA mutase; 5 = isobutyryl CoA dehydrogenase. [Color figure can be viewed at http://wileyonlinelibrary.com]

Five enzymes are involved in the mitochondrial degradation of valine to propionyl‐CoA: isobutyryl‐CoA dehydrogenase, short‐chain enoyl‐CoA hydratase (SCEH), 3‐OH‐isobutyryl‐CoA hydrolase (HIBCH), 3‐hydroxyisobutyric acid dehydrogenase (HIBADH), and methylmalonate semialdehyde dehydrogenase (Figure 1); defects in these enzymes cause inborn errors of valine degradation (Peters et al., 2014; Wanders, Duran, & Loupatty, 2012).

Deficiency of SCEH which converts unsaturated *trans*‐2‐enoyl‐CoA species to the corresponding 3(*S*)‐hydroxyacyl‐CoA (Haack et al., 2015; Peters et al., 2014; Sakai et al., 2015; Tetreault et al., 2015) and deficiency of HIBCH (Brown et al., 1982; Ferdinandusse et al., 2013; Loupatty et al., 2007; Soler‐Alfonso et al., 2015; Stiles et al., 2015) that catalyses the conversion of 3‐OH‐isobutyryl‐CoA to 3‐OH‐isobutyrate, the fourth and fifth steps of valine degradation, respectively, have been associated with LS or Leigh‐like syndrome and deficiencies of multiple mitochondrial respiratory chain enzymes.

Accumulation of toxic methacrylyl‐CoA and acryloyl‐CoA, two highly reactive intermediates that spontaneously react with sulfhydryl groups of, for example, cysteine, and cysteamine, is suspected to cause brain pathology and the biochemical pattern found in HIBCH and SCEH deficiencies (Ferdinandusse et al., 2013; Loupatty et al., 2007; Peters et al., 2014). SCEH also catalyzes the second step of the β‐oxidation of short‐chain fatty acids, that is, the hydration of α,β‐unsaturated enoyl‐CoAs to produce β‐hydroxyacyl‐CoAs (Kanazawa et al., 1993). It has been speculated that it is both the β‐oxidation defect and the block in l‐valine metabolism that contribute to the pathology in SCEH deficiency (Haack et al., 2015). However, Ferdinandusse et al. (2013) presented evidence that despite its broad substrate specificity, SCEH appears to be crucial in valine catabolism, but only of limited importance for mitochondrial fatty acid oxidation and may not play a significant role in isoleucine metabolism.

Peters et al. (2014) described the metabolite *erythro*‐2,3‐dihydroxy‐2‐methylbutyrate in urine of patients with SCEH deficiency. While the exact origin of 2‐methyl‐2,3‐dihydroxybutyrate is currently unknown, we previously noted this metabolite in patients with lactic acidosis of unknown etiology, and in patients with genetically proven metabolic disorders including glycogen storage disease type 1a (OMIM 232200), propionic acidemia (propionyl‐CoA carboxylase deficiency, OMIM 232000), methylmalonic aciduria (methylmalonyl‐CoA mutase deficiency, OMIM 251000), PDH deficiency (OMIM 300502), and multiple carboxylase deficiency due to biotinidase deficiency (OMIM 609019). Despite complete deficiencies of the enzymatic activities in fibroblasts, Ferdinandusse and co‐workers could not detect 2,3‐dihydroxy‐2‐methylbutyrate in the supernatant of patients' cultured fibroblasts (Ferdinandusse et al., 2015). 2,3‐dihydroxy‐2‐methylbutyrate exists as two isomers, *erythro* and *threo*. *Erythro* 2,3‐dihydroxy‐2‐methylbutyrate is elevated in urine of patients with pathogenic *ECHS1* and *HIBCH* variants and propionate defects and clearly segregates when plotted against the *threo* isomer, while the *threo* isomer appears to be dietary related (James Pitt, unpublished observation; Peters et al., 2015).

Given the segregation of *erythro* 2,3‐dihydroxy‐2‐methylbutyrate with metabolic dysfunction, we retrospectively reviewed urine organic acid chromatograms analyzed in the Metabolic Laboratory, Department of Paediatric Laboratory Medicine in Temple Street Children's University Hospital from 2007 to 2010 and identified eight patients with increased *erythro‐*2,3‐dihydroxy‐2‐methylbutyrate without a diagnosis; of these, two were possible candidates for either SCEH or HIBCH deficiencies (Patient 1 and Patient 2). Two siblings of Patient 2 born after 2010 were also included (Patients 3 and 4). We describe four patients from two consanguineous families (Patient 1 from a Pakistani family and Patients 2–4 from an Irish Traveler family) who presented in infancy with LS. All had similar clinical features including developmental delay/regression, faltering growth, hypotonia, seizures, and apneic episodes (Tables 1 and 2). All had previously undergone extensive investigations without reaching a diagnosis.

**Table 1:** Summary of clinical features of four patients with *ECHS1* deficiency

	Patient 1	Patient 2	Patient 3	Patient 4
Gender	Male	Male	Female	Male
Birth history	ECS, 37^+4^ IUGR	First sibling Term baby, SVD	SVD, 37 weeks (sibling RIP at 21 months)	Third affected sibling SVD, 34^+5^
Birth weight	2.6 kg	3.7 kg	3.26 kg	2.31 kg
Age at presentation	5 months	3 months	5 months	2 weeks
Age at death	4 years	21 months	28 months	13 months
Initial presentation	Poor feeding, excessive crying. Head lag, central hypotonia, peripheral hypertonia, Nystagmus Global delay	No neonatal concerns FTT, central hypotonia, hepatosplenomegaly	No neonatal concerns Significant faltering growth, motor delay, central hypotonia, hepatomegaly	Feeding problems, irritable, hypotonic
Seizures	From 11 months	Ocular flutters from 9 months	From 5 months	From 9 months
Faltering growth	Weight 2nd percentile at birth, <0.4th percentile at 1 year, improvement post PEG insertion	At 19 months failure to gain weight at 7.66 kg (<3rd percentile) NG tube inserted at 19 months	5 months OFC at 0.4th percentile; weight at 2nd percentile. NG fed at 15 months, PEG inserted at 20 months	Weight 9th percentile at birth, <0.4th percentile at 12 weeks. NG fed then PEG inserted at 10 months
Global developmental delay and regression	Profound developmental delay with loss of skills	Gross psychomotor delay with loss of skills	Profound developmental delay with loss of skills	Profound developmental delay with loss of skills
Other clinical features	Increased oral secretions, chronic vomiting, apnea	Dyskinetic CP	Dystonic posturing, apnea	Profound irritability, apnea

CP = cerebral palsy; ECS = emergency cesarean section; IUGR = intra uterine growth retardation; MRS = magnetic resonance spectrosopy; OFC = occipital frontal circumference; PEG = percutaneous endoscopic gastrostomy; SVD = spontaneous vaginal delivery.

**Table 2:** Summary of MRI findings

	Patient 1	Patient 2	Patient 3	Patient 4
**T2W hyperintense signal**:				
Globus pallidi	++	++	++	++
Putamina	+	+	+	+
Caudate heads	+	+	+	+
Thalami	−	−	−	+
Cerebral crura	++	++	++	++
Periaqueductal gray matter	−	+	+	+
Pons	+	−	−	+
Cerebellum	−	−	−	+
**Restricted diffusion**:	+	+	+	+
**Brain volume loss**:				
Supratentoria	++	+	++	++
Infratentorial	++	−	+	+
**MRS (Lactate peak)**:	+	+	−	+
**Summary**	Abnormal bilateral signal in the basal ganglia, midbrain, and dendate. Dilation of ventricles, significant progression in cerebral, and cerebellar atrophy. Thinning of corpus callosum.	Abnormal symmetrical bilateral signal in globus pallidus, putamen, head of caudate nuclei, subthalamic nuclei, and cerebral peduncles. Thalamus spared. No atrophy.	Symmetrical abnormal signal in globus pallidi, cerebral crura, caudate nuclei, putamina, lentiform nuclei. Prominent sulci and volume loss.	Focal bilateral symmetrical lesions in thalami, abnormal signal in lentiform nuclei, and caudate nuclei, dilation of ventricles, prominent sulci, and volume loss.

+ Positive, ++ Markedly Positive, − Negative; MRS = magnetic resonance spectroscopy.

## 2 PATIENTS AND METHODS

### 2.1 Patient reports

Patient 1 was the first child (male) born to consanguineous Pakistani parents. He was born by emergency cesarean section at 37 weeks gestation because of intrauterine growth retardation. Birth weight was 2.6 kg (5th percentile). Initially, he was slow to feed and his parents noted there was excessive crying. Global developmental delay was apparent at 5 months of age with central hypotonia, peripheral hypertonia, nystagmus, and failure to visually fix or follow. EEG was normal at 8 months; however, seizures commenced by 1 year of age and proved difficult to control. He was not dysmorphic but had hypertrichosis. Growth was suboptimal with weight less than the 0.4th percentile for age and sex.

At 3 years, he had profound global developmental delay with regression of feeding skills, poor swallow, and chronic vomiting. A percutaneous endoscopic gastrostomy (PEG) tube was inserted to improve feeding which led to some weight gain. Apneic episodes started and became frequent. He had episodes of aspiration pneumonia and he died just before his fourth birthday. His parents subsequently had two further unaffected children (Tables 1 and 2).

Patient 2 was the eldest child (male) born to consanguineous Irish Traveler parents by vaginal delivery at full term following an uneventful pregnancy. Birth weight was 3.7 kg. From 3 months of age motor developmental delay was noted. He had central hypotonia and hepatosplenomegaly. He was failing to thrive and feeding difficulties were noted. At 9 months of age weight was below the 0.4th percentile, head circumference was below the 2nd percentile; he was reviewed by the neurology team for suspected dyskinetic cerebral palsy. He had no dysmorphic features. Gross motor delay was noted while his development was felt to be appropriate in other areas. On examination his spleen tip was palpable and liver was 3 cm below the costal margin. Ocular flutters were noted but no seizures.

His nutrition was supplemented with nasogastric feeds. By 12 months of age he was globally developmentally delayed with regression of motor skills. On examination he showed variable peripheral tone with abnormal mouthing and tongue movements. He continued to have prominent feeding issues and developmental regression. He passed away at 21 months of age. A post mortem was not consented.

Patient 3 was the second child (female) born to this family. Her elder brother (Patient 2) passed away approximately 18 months earlier without a diagnosis at the time of his death. She was born by spontaneous vaginal delivery at 37 weeks gestation and there were no concerns during the pregnancy. She had a good birth weight of 3.26 kg (50th percentile). There were no neonatal concerns initially, but in light of the family history a metabolic screen was performed which showed elevated lactate and abnormal urinary organic acids (Table 3). Her echocardiogram initially showed left ventricular hypertrophy but follow‐up echocardiograms were normal.

**Table 3:** Biochemical results of extensive investigations of four patients with *ECHS1* deficiency

	Patient 1	Patient 2	Patient 3	Patient 4
Plasma lactate (mmol/L)	1.47–>**5.46** (0.6–2.4)	**2.46** (0.6–2.4)	1.50–>**5.68** (0.6–2.4)	1.92–>**4.92** (0.6–2.4)
Plasma alanine (µmol/L)	337–>**764** (176–480)	599 (0–675)	423–>471 (0–675)	383–>**559** (176–480)
Plasma proline (µmol/L)	256–>**717** (89–287)	**468** (0–435)	211 (0–435)	277–>**300** (89–287)
Acylcarnitine analysis (DBS)	Normal profile Slight increase in C_4_OH/isoC_4_OH	Not performed	Slightly decreased free carnitine and long chain AC. C_4_OH/isoC_4_OH not increased	Normal profile C4OH/isoC_4_OH not increased
Organic acid analysis	Mild increases in **3‐MGC** **3‐HIVA** **2,3‐dihydroxy‐2‐methylbutyrate**	Slight increases in **3‐MGC** **2,3‐dihydroxy‐2‐methylbutyrate**	**Marked lactate** Slight increase in **MMA** Mild increase in **3‐MGC** **2,3‐dihydroxy‐2‐** **methylbutyrate**	**Mild dicarboxylic aciduria** Slight increases in **MMA** **3‐MGC** **2,3‐dihydroxy‐2‐methylbutyrate**
PDH activity in fibroblasts	**Reduced activity** No mutations found	Not performed	Normal activity	Not performed
Muscle respiratory Chain complexes (C)	Normal CI, II, II, IV activities	Not performed	**Decreased Complex III activity**	Not performed
Other	*AUH* and multiple single gene tests known to cause LS all negative	*SURF1* genetics was negative	Multiple single gene tests and WES negative	Single gene tests and WES all negative

Results in bold indicate abnormal findings. AC = acylcarnitines; C_4_OH/isoC_4_OH = hydroxybutyryl/hydroxyisobutyryl‐carnitine; DBS = dried blood spot; LS = Leigh syndrome; 3‐MGC = 3‐methylglutaconate; 3‐HIVA = 3‐hydroxyisovalerate; MMA = methylmalonic acid; PDH = pyruvate dehydrogenage activity.

At 5 months of age she showed significant faltering growth; her weight had fallen to the 2nd percentile and her head circumference had fallen from 25th to 0.4th percentile. She was showing signs of motor delay and central hypotonia with moderate head lag. Hepatomegaly was noted at this stage. Seizure activity was first noted at 7 months of age. She developed problems with feeding and was found to be aspirating on thin fluids. By 9 months of age there was significant global developmental delay. Her feeding difficulties prompted insertion of a nasogastric tube. At 15 months her seizure activity had increased consisting of prolonged tonic episodes with tongue protrusion and eye deviation. She was demonstrating abnormal movements and dystonic posturing which were non‐epileptic in nature.

At 20 months of age a PEG feeding tube was inserted. She was noted to be developing apneic episodes of increasing frequency and duration. At 2 years and 3 months of age, she was intubated and ventilated following a respiratory arrest and passed away 11 days later.

Patient 4 was the third affected sibling (male) in this Irish Traveler family. He was born at 34 weeks gestation by spontaneous vaginal delivery. His birth weight was 2.31 kg (9th percentile corrected for gestation). He was noted to have feeding problems with oxygen desaturation and irritability from the outset.

Investigations at 5 weeks of age showed raised lactate and abnormal urinary organic acids, with a similar profile to his sister (Table 3). On review at 12 weeks of age he was found to be irritable, hypotonic and globally developmental delayed. He was not fixing and following and had poor head control. He had significant faltering growth with weight less than 0.4th percentile. At 5 months of age a nasogastric tube was inserted. At 7 months of age he was having increasing feeding difficulties and loose stools. A PEG tube was inserted at 10 months of age.

From 9 months of age seizures were noted which became more frequent and prolonged over the following months. He was admitted to hospital for a “breath holding” episode at 11 months of age. Further developmental regression was noted at this stage; he had become increasingly hypotonic. At 12 months of age he had a significant apneic episode and an upper gastrointestinal bleed. He was intubated and ventilated and had a prolonged hospital stay. He passed away shortly after extubation at 13 months of age.

This patient series involves four patients from two different families. All four patients had similar clinical features and progression. They all had significant developmental delay and central hypotonia was a common early sign. Faltering growth and progressive feeding problems were prominent in all patients. As the clinical syndrome progressed, seizures and apneic episodes became more frequent and severe.

### 2.2 Brain magnetic resonance imagining (MRI) and spectroscopy (MRS)

Patient 1 had a total of three MRI Brain examinations. On the initial imaging at 6 months of age, there was pronounced symmetrical T2W prolongation of the globus pallidi and cerebral crura with corresponding restricted diffusion on apparent diffusion coefficient (ADC). Less marked hyperintensity was evident in the putamen and caudate nuclei. There was a lactate peak on MRS. The thalami, pons, and cerebellum were not involved.

Progressive volume loss was evident on subsequent imaging, most pronounced in the cerebellum. The corpus callosum was markedly thinned on the patient's last imaging at 35 months. There was no restricted diffusion on ADC or an abnormal lactate peak on MRS on the subsequent imaging.

**Figure 2:**
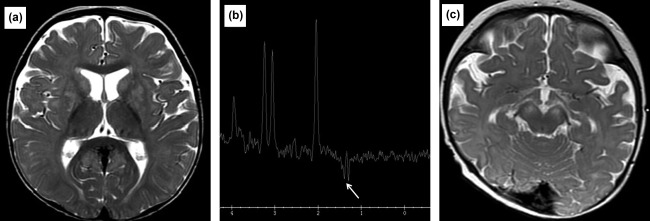
(a) MRI image of Patient 2 at 9 months of age. Axial T2W image at the basal ganglia level demonstrating symmetrical hyperintense foci involving the lentiform nuclei and caudate heads. The thalami are spared. (b) MRS of Patient 2 at 9 months of age. Long TE MRS with left basal ganglia sampling demonstrating a lactate peak at 1.3 ppm. (c) MRI image of Patient 3 at 8 months of age. Axial T2W image at midbrain level, demonstrating bilateral symmetrical hyperintensity of the cerebral crura and subtle hyperintensity of the periaqueductal gray matter

Patient 2 had one single MRI Brain examination at 9 months of age. There was bilateral symmetrical T2W increased signal intensity involving the corpus striatum, cerebral peduncles, and the periaqueductal gray matter. The thalami were not involved. Diffusion weighted imaging was not performed. A lactate peak was present on MRS (Figure 2a,b).

Patient 3 had a total of three MRI brain scans. Her initial MRI at 5 weeks was normal. A subsequent MRI performed at 10 months demonstrated T2W prolongation of the caudate heads, lentiform nuclei, and cerebral crura with corresponding restricted diffusion on ADC. No abnormality was detected on MRS. Generalized brain volume loss was also evident.

There was further progressive brain volume loss, quite marked in the cerebellum on her last MR Imaging at 28 months. There was T2W hyperintensity involving the corpus striatum, which demonstrated volume loss in the interval. There were new areas of restricted diffusion, involving the hypothalamus (Figure 2c). MRS was normal.

Patient 4, the youngest of the three siblings had three MRI brain examinations. The initial MRI at 6 weeks corrected gestational age, showed mild delay of myelination. On subsequent MRI at 10 months of age, there was extensive abnormal symmetrical hyperintensity in caudate nuclei, lentiform nuclei, cerebral crura, periaqueductal gray matter, and bilateral thalami. T2W prolongation was also noted in the posterior tegmental tracts of the medulla.

On his last imaging at 12 months of age, there was marked brain volume loss with prominent sulci and extra‐axial spaces. There was also abnormal T2W hyperintense signal involving the cerebellar gray matter, posterior medulla, periaqueductal gray matter, cerebral crura, thalami, and corpus striata. There were new areas of restricted diffusion seen in the caudate heads. A lactate peak was evident on MRS.

## 3 METHODS

### 3.1 Ethical compliance

Informed consent was obtained from both sets of parents for routine and investigative studies.

Consent was obtained from the parents of family two for whole exome sequencing (WES) of two affected siblings (Patient 3 and Patient 4) according to Temple Street Children's University Hospital procedures. Consent was given to use Patient 2 archived new born blood spot screening card for molecular genetic studies.

### 3.2 Metabolite analysis

Urine *erythro*‐2,3‐dihydroxy‐2‐methylbutyrate was detected as a trimethylsilyl derivative on routine organic acid analysis by standard gas chromatography‐mass spectrometry. Separation from *threo*‐2,3‐dihydroxy‐2‐methylbutyrate was noted and *erythro*‐2,3‐dihydroxy‐2‐methylbutyrate was quantified in urine as described by Peters et al. (2014) in Patient 1 and Patient 4 only.

**Figure 3:**
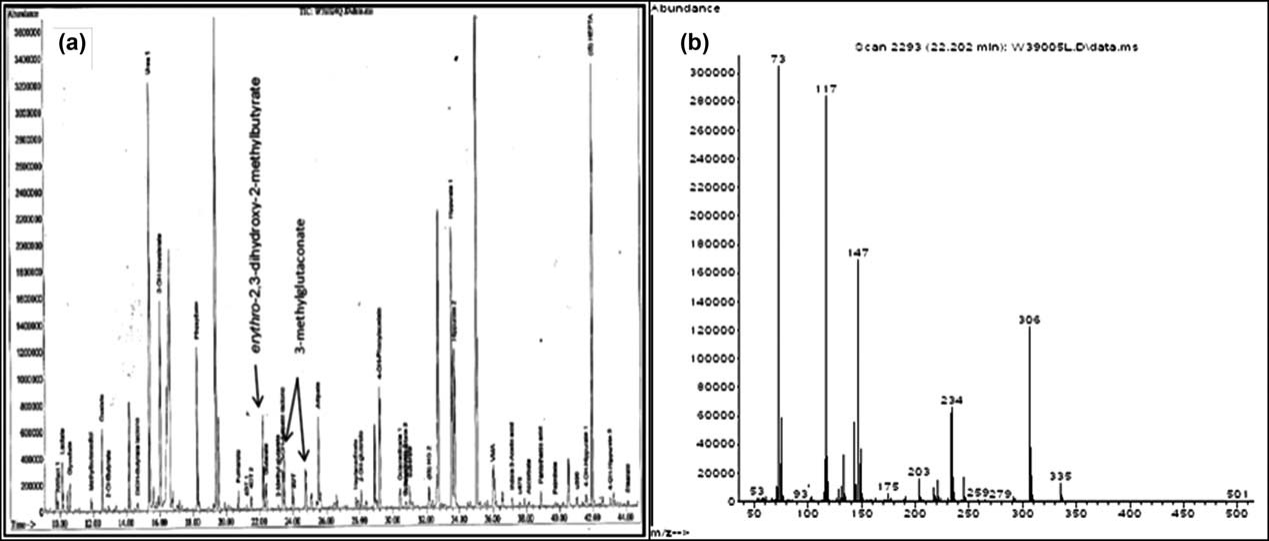
(a) Urine organic acid total ion chromatogram of Patient 1 with ECHS1 deficiency. 3‐hydroxyisovalerate (eluting at 16 min), *erythro*‐2,3‐dihydroxy‐2‐methylbutyrate (eluting at 22.2 min), and 3‐methyglutaconate (2 peaks eluting at approximately 23.5 and 24.5 min) are increased. Internal standards are heptanoylglycine and heptadecanoate (eluting at 32 min and 42 min, respectively). (b) GC/MS Spectra of *erythro*‐2,3‐dihydroxy‐2‐methylbutyrate which elutes separately and just before its *threo* isomer

Spectra for both isomers are similar with notable ion at m/z 306 (Figure 3b); however, they are separated on standard GC/MS organic acid chromatography, with *erythro*‐2,3‐dihydroxy‐2‐methylbutyrate eluting approximately 0.2 minute earlier than *threo*‐2,3‐dihydroxy‐2‐methylbutyrate.

Acylcarnitine analysis of butyl derivatives on dried blood spots (DBSs) was performed by standard tandem mass spectrometry methods on Patients 1, 3, and 4.

Semi‐quantitative urine screening for methacrylate and acrylate derivatives was performed on urine from Patient 1 and Patient 4 only. Analysis was by flow injection tandem mass spectrometry of butyl derivatives as previously described by Peters et al. (2015). Quantitation of urine cysteine, cysteamine, glycine, methacrylyl‐CoA and acryloyl‐CoA conjugates and the separation of 3‐hydroxybutyrylcarnitine (C_4_OH‐carnitine), 3‐hydroxyisobutyryl carnitine (isoC_4_OH‐carnitine), and *erythro*‐2,3‐dihihydroxy‐2‐methylbutyrate is described by Peters et al. (2015).

### 3.3 SCEH enzyme activity measurement

Measurement of SCEH enzyme activity and immunoblotting, were performed in cultured skin fibroblasts from Patient 1 and Patient 3 as described in Peters et al. (2014).

### 3.4 Genetic analyses

All exons and flanking intronic sequences of the *ECHS1* gene of Patient 1 were Sanger sequenced after amplification by PCR from genomic DNA isolated from cultured fibroblasts and using a BigDye Terminator cycle sequencing kit (Applied Biosystems, Foster City, CA).

WES was conducted on Patients 3 and 4 according to standard protocols as previously reported (Besse et al., 2015; Bonnen et al., 2013). Additional di‐deoxy Sanger sequencing analysis of *ECHS1* exon 5 was undertaken using DNA from Patients 2 to 4 (three clinically affected siblings) and their unaffected parents. Molecular genetic investigations for Patient 2, the oldest sibling in Family 2, was undertaken using DNA obtained from his archived newborn bloodspot screening card.

## 4 RESULTS

### 4.1 Laboratory investigations and metabolite results

#### 4.1.1 Patient 1

Initial biochemical investigations showed elevated lactate in both plasma and CSF. There was no significant increase in plasma lactate post feed. Plasma alanine was elevated initially; however, plasma proline was increased on five out of six occasions measured. Other amino acids were essentially normal in plasma and CSF including branched‐chain amino acids (BCAA). Urine organic acid analysis on all four occasions showed abnormal increase in excretion of 3‐methylglutaconate (3MGC) and *erythro*‐2,3‐dihydroxy‐2‐methylbutyrate, with mild ketonuria noted on one sample. Excretion of 3‐hydroxisovalerate was increased slightly on all four occasions but this was not reflected in the acylcarnitine analysis (Figure 3a). The DBS acylcarnitine profile showed a slight increase in signal at m/z 304 hydroxybutyrylcarnitine/hydroxyisobutyrylcarnitine (C_4_OH/isoC_4_OH‐carnitine). This was attributed to C_4_OH‐carnitine, reflecting the mild ketosis noted in urine collected at the same time.

Lactate/pyruvate ratio was measured in perchloric acid de‐proteinised blood and values ranged from 6.9 to 15.2 (reference range 12–20). PDH complex (PDHc) activity in cultured skin fibroblasts (performed as described by Wicking, Scholem, Hunt, and Brown (1986) was slightly decreased at 0.58 nmol/mg protein/min (reference range 0.7–1.1). Analysis of *MT‐ATP8* and *MT‐ATP6* genes associated with LS and analysis of *PDH* genes and *AUH* gene revealed no known or candidate pathogenic variants.

#### 4.1.2 Patient 2

Plasma lactate was marginally increased with essentially normal plasma alanine and proline. Other amino acids were essentially normal including BCAA. Urine organic acids performed on one occasion showed an abnormal increase in excretion of (3MGC) and *erythro*‐2,3‐dihydroxy‐2‐methylbutyrate. DBS acylcarnitines were not analyzed. He had no mutations in the *SURF1* gene. Skin or muscle biopsies were not consented and no further investigations were performed prior to death (Table 3).

#### 4.1.3 Patient 3

Plasma lactate increased with progression of disease while plasma alanine and proline levels remained within reference range. Other amino acids were essentially normal including branched‐chain amino acids. There was a significant increase in plasma lactate from 1.71 to 3.39 mmol/L (reference range 0.6–2.4 mmol/L) 60 min post ingestion of a glucose load. Urine organic acids performed on two occasions showed an abnormal increase in excretion of 3‐MGC and *erythro*‐2,3‐dihydroxy‐2‐methylbutyrate, and a marked lactate excretion reflecting increased plasma lactate at time of collection. There was a slight increase in methylmalonic acid (MMA) excretion on the initial urine organic acid analysis which normalized on subsequent testing. DBS acylcarnitine analysis revealed a slightly low free carnitine with slightly decreased long chain acylcarnitines, probably reflecting poor feeding, and low carnitine intake. There was no increase in C_4_OH/isoC_4_OH‐carnitine nor methylmalonyl/succinyl‐carnitine (C_4_DC) making either succinyl‐CoA ligase ADP forming β‐subunit (SUCLA2) or succinate‐CoA‐ligase α‐subunit (SUCLG1) deficiency unlikely. Both of these conditions are associated with elevated plasma lactate, increased MMA and basal ganglia involvement on neuroimaging (Carrozzo et al., 2007; Ostergaard et al., 2010).

PDH activity and fatty acid β‐oxidation studies in skin fibroblasts was normal. The activities of respiratory chain complexes I, II, and IV were normal within a frozen muscle homogenate although the activity of complexes II + III (representing complex III) was found to be decreased in her skeletal muscle. The patient did not have the common mitochondrial DNA mutations. Similarly, she did not have the common pathogenic *POLG* variants associated with Alpers' syndrome. A novel m.12173T > C variant of uncertain pathogenicity within the *MTTH* gene (encoding mt‐tRNA^His^) was identified but further analyses were not pursued as it did not segregate within the family with a clinically affected status. Analysis of large‐scale mtDNA rearrangements demonstrated no detectable mtDNA deletions or duplications and WES yielded no candidate variants (Table 3).

#### 4.1.4 Patient 4

Plasma lactate levels increased with disease progression while plasma alanine and proline levels remained within reference range similar to those of his sister though they were only measured in the neonatal period. Other amino acids were also normal including branched‐chain amino acids. Initial urine organic acids performed showed an abnormal increase in excretion of 3‐MGC similar to his siblings and was persistent in all six urines. There was a slight increase in excretion of MMA in the second and third samples which normalized with later testing. A mild dicarboxylic aciduria was present in one profile reflecting lipolysis, probably secondary to resolving ketosis. Excretion of *erythro*‐2,3‐dihydroxy‐2‐methylbutyrate while detectable in all six urines was not considered significant enough for comment on qualitative analysis during earlier sampling and was only considered to be increased when he was approximately 5 months of age. The acylcarnitine profile was normal and neither C_4_OH/isoC_4_OH‐carnitine nor methylmalonyl/succinyl‐carnitine (C_4_DC) were increased. As with his two siblings, analysis of mtDNA genes commonly associated with LS and WES did not identify a genetic cause. Skin and muscle biopsies were not consented (Table 3).

All four patients had overlapping biochemical abnormalities; elevated plasma lactate, with or without increased plasma alanine and proline, 3‐methylglutaconic aciduria with elevated *erythro*‐2,3‐dihydroxy‐2‐methylbutyrate and usually normal C_4_OH/isoC_4_OH‐carnitine and normal branched‐chain amino acid levels.

Quantitative analysis of *erythro*‐2,3‐dihydroxy‐2‐methylbutyrate confirmed that levels were significantly increased in the urine of Patient 1 and Patient 4 (Table 3). Excretion of 3‐MGC was increased in all samples from all four patients. Notable was also the mild excretion of methylmalonic acid, a propionate metabolite downstream from SCEH and HIBCH on the valine degradation pathway, in Patients 3 and 4 and mildly increased excretion of 3‐hydroxyisovalerate, a leucine metabolite, in all four samples from Patient 1. None of these metabolites were reflected in acylcarnitine analysis. Propionylcarnitine (C_3_), methylmalonyl/succinyl‐carnitine (C_4_DC), and hydroxyisovaleryl‐carnitine (C_5_OH) were all within the reference range in three of the patients where acylcarnitine profiling was performed. Patient 1 had mild ketonuria in one of his four urines and Patient 4 had one urine which was mildly lipolytic; however, neither he nor the other three patients showed any markers of impaired mitochondrial β‐oxidation in either urine organic acid profile or acylcarnitine profile on DBS analysis where samples were available.

LC‐MS/MS analysis (Peters et al., 2015) of urine from Patients 1 and 4 revealed increased excretion of methacrylyl‐CoA and acryloyl‐CoA related metabolites without significant increases of isoC_4_OH‐carnitine; this metabolic profile was highly suggestive of SCEH deficiency (Table 4).

**Table 4:** Urine metabolite results (µmol/mmol creatinine) LC‐MS/MS in patients with *ECHS1* deficiency

					Controls	
LC‐MS/MS	Range	*n*	Patient 1	Patient 4	*ECHS1* positive	*HIBCH* positive
Acryloyl cysteamine	<1.2	19	2.0	2.6	6.5	12.2
Acryloyl‐l‐cysteine	<2.2	19	1.9	2.3	5.7	3.8
*N*‐acetyl‐acryloyl‐cysteine	<9.0	9	15.6	13.1	43.4	10.0
Methacryl‐cysteamine	<10	19	8.6	9.4	23.4	13.0
Methacryl‐l‐cysteine	<5.3	19	11.9	9.7	32.0	26.6
*N*‐acetyl methacryl‐cysteine	<0.4	9	3.9	3.0	10.8	6.6
*S*‐(2‐carboxypropyl)cysteine carnitine ester (UMS/MS)	<0.4	334	1.1	1.1	25.2	5.3
3‐hydroxyisobutyryl carnitine(isoC_4_OH)	<0.38	9	0.48	0.2	0.1	6.79
*Erythro*‐2,3‐dihydroxy‐2‐methylbutyrate (GC/MS)	<25	48	492	322	2,370	704

### 4.2 SCEH enzyme activity measurement and immunoblotting

SCEH enzyme activity was measured in the fibroblasts of Patients 1 and 3 only. SCEH enzyme activity was markedly reduced in fibroblasts of Patient 1 (40 nmol/(min.mg protein)) and Patient 3 (27 nmol/[min.mg protein]) compared to the reference values (199–670 nmol/[min.mg protein]). Immunodetection of steady‐state SCEH protein in fibroblast homogenates of Patient 1 and 3 by western blotting revealed the absence of the 27 kDa band corresponding to SCEH (Ferdinandusse et al., 2015).

### 4.3 *ECHS1* genetic analyses

#### 4.3.1 Family 1

Patient 1 was apparent homozygous for a previously reported c.476A > G, p.(Gln159Arg) variant (Haack et al., 2015) in exon 4 of the *ECHS1* gene (GenBank Accession Number: NM_004092.3) corroborating the enzymatic diagnosis of SCEH deficiency. The c.476A > G, p.(Gln159Arg) *ECHS1* variant has been previously reported in either the homozygous or compound heterozygous state in clinically affected patients (Haack et al., 2015). Unfortunately, parental DNA samples were not available to confirm the apparent homozygosity in Patient 1.

#### 4.3.2 Family 2

**Figure 4:**
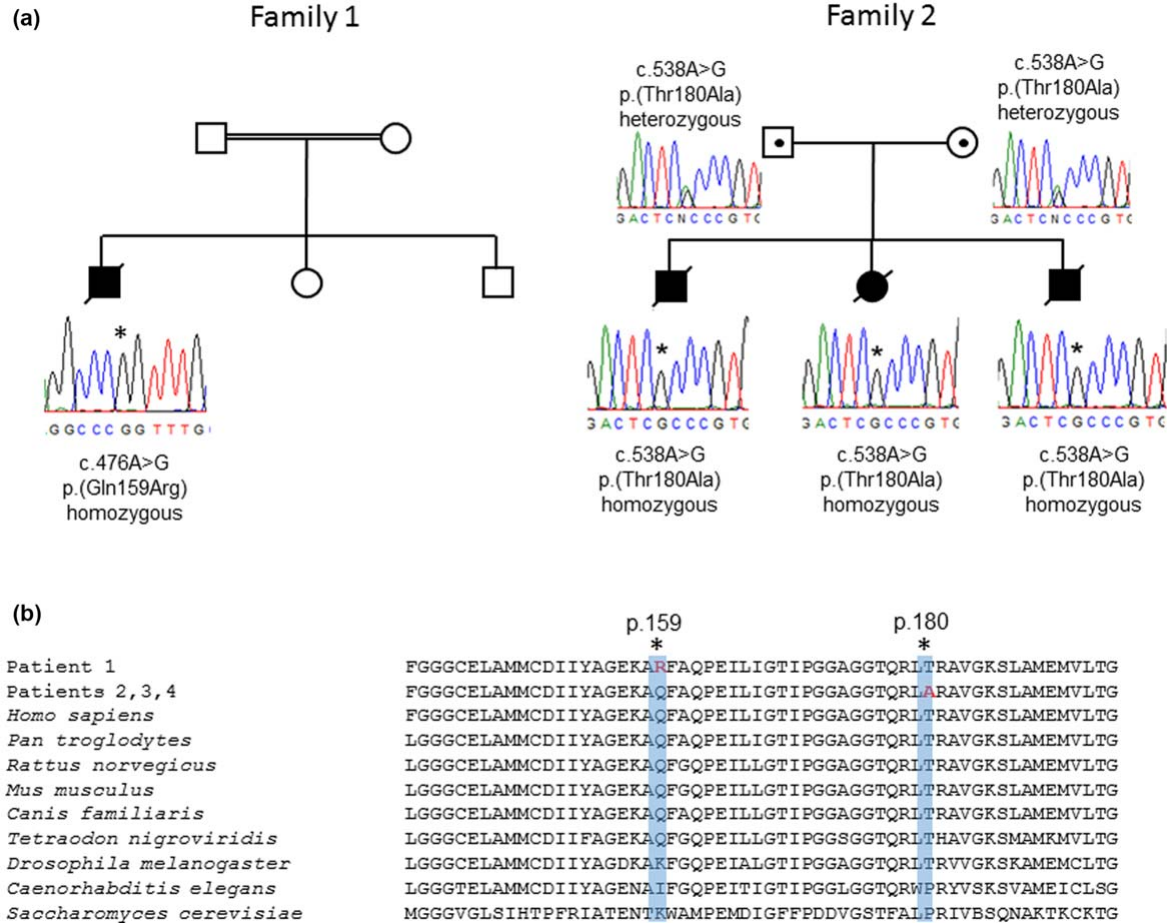
(a) Sequencing chromatograms showing the homozygous c.476A>G, p.(Gln159Arg) ECHS1 variant in Family 1 and the c.538A>G, p.(Thr180Ala) ECHS1 variant in Family 2. (b) Conservation studies support the evolutionary importance of the p.Gln159 and p.Thr180 residues. [Color figure can be viewed at http://wileyonlinelibrary.com]

Initial WES analysis of Patients 3 and 4 did not identify any potentially pathogenic variants, but following a biochemical diagnosis of SCEH deficiency in Patient 3, reanalysis of the exome data at the *ECHS1* locus revealed exon 5 was not captured in this exome assay, and had no corresponding sequencing data, despite an average 100× coverage exome‐wide. Sanger sequencing of exon 5 identified a previously reported homozygous c.538A > G, p.(Thr180Ala) *ECHS1* variant in Patients 2, 3, and 4 (Tetreault et al., 2015). Analysis of parental samples confirmed recessive inheritance of the c.538A > G, p.(Thr180Ala) *ECHS1* variant in this family (Figure 4).

## 5 DISCUSSION

This case series was part of a retrospective study performed following the publication that the presence of *erythro*‐2,3‐dihydroxy‐2‐methylbutyrate in urine had been identified in some patients with SCEH and HIBCH deficiency. This review involves four patients from two families where no definitive cause of death was made at the time of death in two of the index cases in each family.

All four patients were diagnosed with SCEH deficiency and had similar clinical features and progression. They all had significant developmental delay. Central hypotonia was a common early sign. Faltering growth and progressive feeding problems were prominent in all cases. As the clinical syndrome progressed, seizures and apneic episodes became more frequent and severe. Neuroimaging of all four patients showed progressive bilateral symmetrical lesions in basal ganglia in keeping with LS (Baertling et al., 2014; Leigh, 1951). Cerebral and cerebellar atrophy and a lactate doublet peak on MRS of the left basal ganglia was present in three out of the four patients. All four patients had overlapping biochemical abnormalities; elevated plasma lactate, with or without increased plasma alanine and proline, 3‐methylglutaconic aciduria (3‐MGA) with elevated *erythro*‐2,3‐dihydroxy‐2‐methylbutyrate and normal hydroxyisoC_4_OH‐carnitine and branched‐chain amino acid levels.

Increased urine excretion of methacrylyl‐CoA and acryloyl‐CoA related metabolites analyzed by LC‐MS/MS pointed to SCEH deficiency. SCEH enzyme activity was markedly reduced in fibroblasts and both families harbor homozygous *ECHS1* mutations.

All four patients had increased excretion of *erythro*‐2,3‐dihydroxy‐2‐methylbutyrate; this metabolite derived from acryloyl‐CoA was originally described in SCEH deficiency in 2014 by Peters et al. Latter patient reports document concentrations of varying magnitude (Bedoyan et al., 2017; Ferdinandusse et al., 2015; Peters et al., 2015) and that levels may be unreliable shortly after birth (Ganetzky et al., 2016) which was the case in Patient 4. Two out of four patients had increased methylmalonic acid which has been reported previously in a patient with SCEH deficiency (Tetreault et al., 2015).

All four of patients had consistent elevated levels of 3‐MGC detected in their urine samples and Patient 1 had elevated 3‐HIVA although his *AUH* gene sequencing was negative. In primary 3‐MGA, deficiency of 3‐methyl glutaconyl Co A hydratase due to mutations in the *AUH* gene are directly responsible for the accumulation of 3MGC while in secondary 3MGA, no defect in leucine catabolism exists and the metabolic origin of 3MGC is unknown (Su & Ryan, 2014). Increased 3‐HIVA has been previously reported in a 1‐year‐old male patient (Ferdinandusse et al., 2015) and increased excretion of 3‐MGC has been reported previously in at least one case of SCEH deficiency, a 7‐year‐old female and may represent a later biochemical feature in milder cases (Ferdinandusse et al., 2015) and noted to be increased transiently following long‐term follow‐up (Huffnagel et al., 2017). All of our patients were from consanguineous unions so it is possible that excretion of 3‐MGC is not related to *ECHS1* mutations but to a separate condition or secondary to mitochondrial dysfunction.

There is a rapidly growing group of IEMs with a syndromic phenotype reviewed by Wortmann, Kluijtmans, Engelke, Wevers, and Morava (2012) and Wortmann et al. (2013) causing secondary 3‐methylglutaconic aciduria (3‐MGA) due to defective phospholipid remodeling or mitochondrial membrane associated disorders. The excretion of 3‐MGC can be highly variable or intermittently normal in secondary 3‐MGA and is generally less than levels seen in primary 3‐MGA due to *AUH*. Clinical features of secondary 3‐MGA are heterogeneous but distinctive, rare, but highly characteristic, neurometabolic syndromes (Saunders et al., 2015; Wortmann et al., 2013, 2015).

3‐MGC is also is a marker not only for mitochondrial dysfunction in general, but for specific mitochondrial disorders (Mandel et al., 2016; Oláhová et al., 2017; Wortmann et al., 2013).

Our patients had extensive mitochondrial molecular genetic studies performed. WES data showed no evidence for an alternative etiology in the second family so it is less likely that 3‐MGC excretion is due to any of the reported conditions causing secondary 3‐MGA and more likely secondary to non‐specified mitochondrial dysfunction. Furthermore, we are aware of 4 out of 4 *ECHS1* deficient patients from Melbourne with persistently increased 3MGC excretion (unpublished observations and personal communications), indicating that ECHS1 deficiency should be added to the growing list of causes of 3MGA.

Secondary PDHc and respiratory chain complex deficiencies have been reported with SCEH deficiency, as previously stated. Only Patient 1 and Patient 3 had muscle biopsies performed, showing a mild secondary PDH deficiency in Patient 1 while Patient 3 had reduced complex III activity, which would support published data (Bedoyan et al., 2017; Ferdinandusse et al., 2013; Sakai et al., 2015).

A wide phenotypic spectrum is now emerging for SCEH deficiency, ranging from lethality in the first days of life to adult patients who may not fulfill all criteria for LS. Urine metabolite levels correlate with clinical severity and specific separation of isoC4OH and OH‐C4‐carnitine isomers can distinguish between SCEH and HIBCH deficiency (Al Mutairi et al., 2017; Bedoyan et al., 2017; Ganetzky et al., 2016; Haack et al., 2015; Nair et al., 2016).

The increased excretion of methacrylyl‐CoA, acryloyl‐CoA adducts, and *erythro*‐2,3‐dihydroxy‐2‐methylbutyrate in the two patients quantitated by LC‐MS/MS are consistent with SCEH deficiency; however, they are less than those previously reported in clinically severe cases (James Pitt, unpublished observations; Peters et al., 2014, 2015). Evidence is emerging that metabolite levels may correlate with disease severity, being subtle or normal for some metabolites in clinically milder cases (Haack et al., 2015; Yamada et al., 2015) and retrospective analysis of *S*‐(2‐carboxypropyl)cysteamine, *S*‐(2‐carboxypropyl)cysteine, and *N*‐acetyl‐*S*‐(2‐carboxypropyl) cysteine can be a diagnostic clue in the disease spectrum of ECHS1 deficiency (A Mutairi et al., 2017). SCEH activity in cultured fibroblasts was markedly reduced but not completely deficient, which is in agreement with a milder clinical phenotype considering the age of death when compared to some other published cases (Bedoyan et al., 2017; Haack et al., 2015; Peters et al., 2014). Ferdinandusse et al. investigated the role of SCEH in fatty acid and branched‐chain amino acid metabolism in four patients with mutations in *ECHS1* and results from their enzyme activity measurements and immunoblot analysis strongly suggest that there is a correlation between the residual SCEH enzyme activity and the severity of the clinical symptoms (Ferdinandusse et al., 2015). In addition, excretion of 2,3‐dihydroxy‐2‐methylbutyrate may not have been reported or checked in some historical cases because its significance was not known at the time; it may be a more common biochemical feature of SCEH deficiency than we are aware of.

The underlying genetic cause of the SCEH deficiency observed in Patient 1 is a previously reported homozygous c.476A > G (p.Gln159Arg) pathogenic *ECHS1* variant associated with functional SCEH enzyme deficiency, thereby confirming the clinical diagnosis. Of interest, Family 3 in a previously reported cohort of patients with *ECHS1*‐related disease (Haack et al., 2015) is also of Pakistani origin, raising the possibility that the p.(Gln159Arg) variant may represent a founder mutation in this population.

The c.538A > G, p.(Thr180Ala) *ECHS1* homozygous variant identified in Family 2 (Patients 2, 3, and 4) has been previously reported with the suggestion that it may represent a single French‐Canadian ancestral mutation (Tetreault et al., 2015). Follow‐up data presented here supports a shared haplotype involving the reported French‐Canadian cases and our Irish Traveler family, thus we propose this variant has an Irish ancestral origin with subsequent migration to Canada.

Canadian immigration history dates back to the 17th century when the land was colonized first by the French in Quebec and then by the British in Newfoundland. Many thousand men from the South East of Ireland where Family 2 originates played their part in early Newfoundland history. The Irish arrived in more significant numbers in the 1760s and another sizeable group of Irish immigrants arrived in 1823–1825. In 1847 alone at the time of The Great Irish Famine close to 100,000 arrived in Grosse Isle, an island in present day Quebec. Family 2 are members of the Irish Travelling Community. Gilbert, Carmi, Ennis, Wilson, and Cavalleri (2017) demonstrated evidence for population substructure within the Irish Traveler population, and estimate a time of divergence from the settled Irish before the Great Famine of 1845–1852.

Genotypes analysis of SNPs surrounding the c.538A > G, p.(Thr180Ala) *ECHS1* mutation were extracted from the WES data for Patients 3 and 4 and compared to data from the original Tetreault et al. (2015) study (Supporting Information Table S1) which showed a common haplotype spanning at least 1,200 Mb around the *ECHS1* locus, supporting a common founder.

WES has accelerated the discovery of new genes and pathways involved in LS, providing novel insights into the pathophysiological mechanisms. No general curative treatment is available for this devastating disorder, although several recent studies imply that early treatment might be beneficial for some patients depending on the gene or process affected, for example, the ketogenic diet has been noted to be helpful in patients with PDHc deficiency and some patients with PDHc deficiency and LS may show small benefits from vitamin or co‐factor supplementation with coenzyme Q10, thiamine or riboflavin (Baertling et al., 2014; Gerards, Sallevelt, & Smeets, 2016; Sofou et al., 2014). Mahajan, Constantinou, and Sidiropoulos (2017) describe a case of ECHS1 deficiency‐associated paroxysmal exercise‐induced dyskinesias with initial symptomatic improvement after 3 months treatment with a mitochondrial cocktail. Soler‐Alfonso et al. (2015) proposed that valine restriction made significant improvement in bilateral ptosis and postural tone of their one HIBCH deficient patient when plasma valine was lowered to 83 µmol/L (reference range 82–293). However, valine restriction alone may not be effective because it was shown that it did not decrease valine levels in rat brain (Hutchison, Zarghami, Cusick, Longenecker, & Haskell, 1983). Restricting leucine, isoleucine, and valine (i.e. a Maple Syrup Urine Disease diet) does decrease brain valine levels so this approach may show better benefits. CSF valine is normal in SCEH/HIBCH deficiency, and the methacrylyl‐CoA and acryloyl‐CoA toxic metabolites are three steps downstream in the valine pathway. Therefore, decreasing cerebral valine may not make a significant impact on the accumulation of toxic metabolites. Treatment with cysteamine and/or *N*‐acetylcysteine is both safe and widely used and cross the blood brain barrier. Some benefits have been shown in Huntington's disease, Parkinson's disease and Alzheimer's disease, some aspects of which involve glutathione depletion, by enhancing natural detoxification (Besouw, Masereeuw, van den Heuvel, & Levtchenko, 2013). Currently, there is no direct evidence of cerebral glutathione depletion in *ECHS1* or *HIBCH* gene defects. However, a fully functional glutathione system may still not be efficient enough to prevent damage from accumulating methacrylyl‐CoA and treatment with cysteamine or *N*‐acetylcysteine may be a useful adjunct to the detoxification of these metabolites.

## 6 CONCLUSION

The presence of detectable *erythro*‐2,3‐dihydroxy‐2‐methylbutyrate in urine is a nonspecific biochemical finding. 3MGA is not a discriminative feature but a minor finding in the biochemical phenotype of ECHS1 deficiency; however, in combination with increased excretion of *erythro*‐2,3‐dihydroxy‐2‐methylbutyrate, elevated plasma lactate and normal acylcarnitine profile in a patient with LS should prompt consideration of SCEH deficiency.

It is a known limitation of WES that not all nucleotides of every coding exon are captured. This can be due to the nature of the primary sequence, for example GC‐rich and repetitive regions are not well ascertained by hybridization‐based capture assays. Additionally, multiple different gene annotation systems exist that do not show complete agreement and capture assays have been designed according to best approximations of genic structures. In this study, WES was conducted to an exome‐wide average coverage of 100x, however, there were no reads present in *ECHS1* exon 5. This underlines the importance of taking into account coverage when interpreting exome results.

Our report also highlights that biochemical analyses remain the gold standard for the diagnosis of some patients with inborn errors of metabolism and, where possible, a molecular genetic diagnosis should always be contextualized with functional (e.g. biochemical) data.

## WEB RESOURCES

aGVGD: http://agvgd.hci.utah.edu/agvgd_input.php

Mutation Taster: http://www.nature.com/nmeth/journal/v11/n4/full/nmeth/journal/v11/n4/full/nmeth.2890.html

Polyphen‐2: http://genetics.bwh.harvard.edu/pph2/index.shtml

Online Mendelian Inheritance in Man (OMIM): http://www.ncbi.nlm.nih.gov/omim/

SIFT: http://sift.jcvi.org/

Canadian Immigration History: the arrival of Irish Immigrants: http://www.irish-genealogy-toolkit.com/canadianimmigrationhistory

Irish Genealogy and Family History—Library and Archives Canada: http://www.bac-lac.gc.ca/immigration

## CONFLICT OF INTEREST

The author(s) declare that there is no conflict of interest regarding the publication of this article.

## Supporting information

Additional Supporting Information may be found online in the supporting information tab for this article.

Supporting Information
