# Supplementary material for: Clinical, biochemical, and genetic features of four patients with short‐chain enoyl‐CoA hydratase (ECHS1) deficiency
Source: Am J Med Genet A. 2018 Mar 25;176(5):1115–27. doi: 10.1002/ajmg.a.38658 (PMC5947294; doi:10.1002/ajmg.a.38658)
Supplement: Supplementary file 1 — Supporting Information [file AJMG-176-1115-s001.docx]

**Supplemental Table 1:** SNP analysis of patients with the c.538A>G, p.Thr180Ala *ECHS1*variant reveals a common haplotype spanning at least 1200Mb, shown in blue. Patient 2 and Patient 3 (sibs, family 2) share a common c.538A>G, p.Thr180Ala *ECHS1* allele with the reported compound heterozygous cases (shown here as R1-R4) (Tetreault et al., 2015). R1-R4 data included with permission from Drs Tetreault and Geraghty.

|  | **SNP ID** | **rs2474328** | **rs2804003** | **rs12781609** | **rs9418953** | **rs3008326** | **rs3008334** | **rs34780987** | **rs2230261** | **p.Thr180Ala** | **rs1049951** | **rs1046178** | **rs3737031** |
| --- | --- | --- | --- | --- | --- | --- | --- | --- | --- | --- | --- | --- | --- |
|  | **Position (hg19)** | **134161633** | **134598628** | **134748331** | **135053773** | **135087521** | **135098672** | **135165623** | **135180430** | **135180474** | **135184126** | **135205009** | **135368590** |
| **P2** | allele1 | **G** | **G** | **C** | **A** | **A** | **A** | **A** | **G** | **C** | **A** | **G** | **C** |
|  | allele2 | **G** | **G** | **C** | **A** | **A** | **A** | **A** | **G** | **C** | **A** | **G** | **C** |
| **P3** | allele1 | **G** | **G** | **C** | **A** | **A** | **A** | **A** | **G** | **C** | **A** | **G** | **C** |
|  | allele2 | **G** | **G** | **C** | **A** | **A** | **A** | **A** | **G** | **C** | **A** | **G** | **C** |
| **R1** | allele1 | **G** | **G** | **C** | **G** | **G** | **G** | **G** | **G** | **T** | **A** | **G** | **T** |
|  | allele2 | **G** | **G** | **C** | **A** | **A** | **A** | **A** | **G** | **C** | **A** | **G** | **C** |
| **R2** | allele1 | **G** | **A** | **T** | **G** | **A** | **A** | **G** | **G** | **T** | **A** | **G** | **C** |
|  | allele2 | **G** | **G** | **C** | **A** | **A** | **A** | **A** | **G** | **C** | **A** | **G** | **C** |
| **R3** | allele1 | **G** | **A** | **T** | **G** | **A** | **A** | **G** | **G** | **T** | **A** | **G** | **C** |
|  | allele2 | **G** | **G** | **C** | **A** | **A** | **A** | **A** | **G** | **C** | **A** | **G** | **C** |
| **R4** | allele1 | **G** | **G** | **C** | **A** | **A** | **A** | **G** | **G** | **T** | **A** | **G** | **T** |
|  | allele2 | **G** | **G** | **C** | **A** | **A** | **A** | **A** | **G** | **C** | **A** | **G** | **C** |
